# A powerful weighted statistic for detecting group differences of directed biological networks

**DOI:** 10.1038/srep34159

**Published:** 2016-09-30

**Authors:** Zhongshang Yuan, Jiadong Ji, Xiaoshuai Zhang, Jing Xu, Daoxin Ma, Fuzhong Xue

**Affiliations:** 1 Department of Biostatistics, School of Public Health, Shandong University, Jinan 250012, China; 2 Department of hematology, Qilu hospital of Shandong University, Jinan 250012, China

## Abstract

Complex disease is largely determined by a number of biomolecules interwoven into networks, rather than a single biomolecule. Different physiological conditions such as cases and controls may manifest as different networks. Statistical comparison between biological networks can provide not only new insight into the disease mechanism but statistical guidance for drug development. However, the methods developed in previous studies are inadequate to capture the changes in both the nodes and edges, and often ignore the network structure. In this study, we present a powerful weighted statistical test for group differences of directed biological networks, which is independent of the network attributes and can capture the changes in both the nodes and edges, as well as simultaneously accounting for the network structure through putting more weights on the difference of nodes locating on relatively more important position. Simulation studies illustrate that this method had better performance than previous ones under various sample sizes and network structures. One application to GWAS of leprosy successfully identifies the specific gene interaction network contributing to leprosy. Another real data analysis significantly identifies a new biological network, which is related to acute myeloid leukemia. One potential network responsible for lung cancer has also been significantly detected. The source R code is available on our website.

Complex disease is rarely caused by a single biomolecule (e.g. protein, metabolite), but reflects various pathobiological processes interacting in a complex network^1^. Numerous risk factors that are related to a disease often act together through networks controlling the disease occurrence, development and prognosis. It would inevitably lose information to analyze the individual component only. In fact, one single factor can express some certain effects on a disease when studying it alone, while this effect could change substantially when studying it within one system or network, and vice versa^2^. Therefore, biomolecules should not be studied beyond the biological systems or networks they are involved in ref. 3. In biological networks, the nodes often represent biomolecules (e.g., genes and proteins), and the edges represent functional, causal or physical interactions between the nodes. An appealing feature of the network is its ability to visualize the topology structure among biological components and improve the understanding of their complex interplays and interconnections. From the perspective of network medicine, different physiological conditions may manifest as different biological networks. Statistical comparison of group differences between biological networks can provide new insight into the underlying disease mechanism, and can have extensive biomedical applications^4^,^5^,^6^,^7^,^8^. For instance, it could provide statistical evidence to give the significant pathways priority for drug targets, which will undoubtedly shorten the time required for drug development, hence saving potential cost.

From the epidemiological perspective, traditional epidemiology has suffered from increasing criticism partly because it often pays more attention to the identification of a single risk factor than the network that is related to a disease, which makes it difficult to deeply explore disease mechanism^9^. With the development of recent technological advances in high-throughput omics platforms, some researchers suggested to integrate various omics data with traditional epidemiology, and further create a network system to study the underlying disease mechanisms in breadth and depth at the human population level. It successfully promotes the emergence of systems epidemiology^9^,^10^. The essential task is still to identify which network. rather than single factor, can affect the different physical conditions (e.g. patients and healthy controls).

Statistical methods are in great need to detect group differences between biological networks. Thus far, several methods have been developed to utilize network topology information to explore various biomedical phenomenons. Langfelder *et al*.^11^ proposed several measures to compare network topologies for weighted correlation networks. Chen *et al*.^12^ used an additive element-wise score to compare a gene regulatory network estimate to a known network. Zhang *et al*.^13^ provided a differential dependency network analysis to detect topological changes in transcriptional networks between subclasses of breast cancer. Yates *et al*.^14^ developed an additive element-wise-based dissimilarity measure for biological network hypothesis tests. However, most of these methods mainly focus on the difference of network topology and have limited ability to capture the changes in nodes. Although the difference of single node may be weak, the aggregated differences of several nodes can be quite strong. It will inevitably lose efficiency to only consider the difference of connection, while omitting the differences of nodes. Recent network comparison methods can be classified into two major categories^15^. One is alignment-based methods, which aim to find a mapping between the nodes of two (or more) networks that preserves many edges and a large subgraph between the networks. The other is alignment-free methods, which aim to quantify the overall topological similarity between networks, they are computationally less expensive than alignment-based methods, and produce a score that quantifies the overall similarity between the two networks. Currently, the best alignment-free network comparison method is Graphlet Correlation Distance^16^,^17^. which was shown to be the most accurate in clustering topologically similar networks, the most noise-tolerant and the most computationally efficient. Nevertheless, the main purpose of these methods is how well to group or cluster topologically similar networks, and most of them mainly focus on undirected networks, while a large set of interesting biological networks such as metabolic, cell signaling or transcriptional regulatory networks are intrinsically directional. Recently, Ji *et al*.^18^ developed a statistical test for detecting the pathway effect contributing to disease under the framework of systems epidemiology. Yet it is limited to the pathway with chain structure, and can only capture changes in the edges while ignoring the changes in the nodes.

One directed biological network usually involves nodes to symbolize biological components and arrows to represent their relationships, which cannot be simply signified by the correlation coefficient commonly used in undirected networks. For instance, the directed edges can reflect the exact nature of mutual regulation mechanisms (promote or suppress) among genes in regulation network, and a cellular signaling network can be used to describe various interactions of proteins in human cells. Generally, both changes in the nodes (e.g. the magnitude of each gene’s expression change), and changes in the edges (e.g. the strength of regulation) can lead to the whole network difference. Even with the same magnitude of edges, it should also be claimed that two networks are different if reverse direction of edges exist. Therefore, the network difference is far from the simple summation of changes in the nodes and changes in the edges, and the network topology structure cannot be ignored since it can at least provide us the relative position of nodes.

In the present study, we develop a new statistical test for detecting group differences between directed biological networks, which is independent of the network attributes and can, in principle, capture the changes in nodes and edges, as well as simultaneously accounting for the topology structure through putting more weights on the difference of nodes locating on relatively more important position in the network. Various simulations have been conducted to assess the performance of the proposed method, under the network has the same or different structure between the two groups, respectively. Three real data sets were further analyzed to evaluate its performance in practice.

## Methods

**Figure 1:**
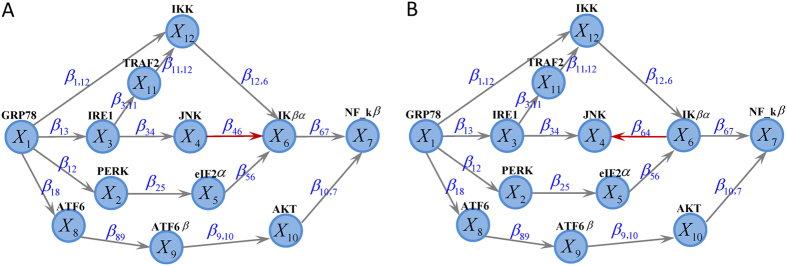
The existed network generated from three branches of the unfolded protein response under sever endoplasmic reticulum stress (A) and the imagined network with reverse direction between *X*_4_ and *X*_6_ (B).

We denote the two directed networks in the two groups (e.g., cases and controls) by *G*^*D*^ and *G*^*C*^, and the sample size is *n*_1_ and *n*_2_, respectively, the null hypothesis test is that no difference exists between *G*^*D*^ and *G*^*C*^. Let *V*(*G*^*D*^) and *E*(*G*^*D*^) denote the set of all nodes and directed edges in *G*^*D*^, the node 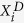 represents general biomolecule such as gene expression level, protein and metabolite. 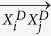 indicates the directed edge 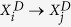 (*i* ≠ *j*, 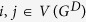), 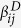 represents the effect of 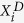 on 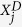 if 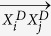 exist (e.g. the regulation strength of 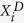 on 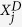). Note that 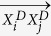 and 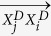 is different. Let 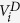 denote the number of children nodes for 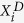, 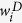 as the relative weight for 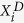, define 
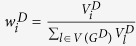
. That is to say, the relative weight for a node is defined as the proportion of the number of its children nodes among the number of children from all network nodes, where the number of children nodes for each node variable is calculated by exhaustively visiting its connected nodes with downstream direction. Let 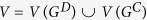, 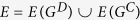, we propose the weighted nodes and edges statistic (*WNES*) as


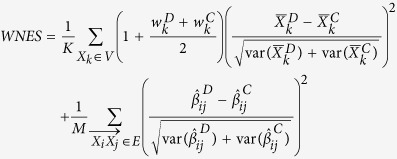


where 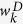, 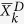 and 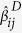 indicate the relative weight, the sample mean and the estimates of 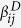 in 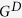, 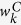, 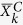 and 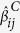 are the corresponding quantities in 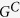. Note that network structure (including the direction of edges) in 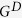 may be different from that in 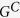, *K*and *M* is the number of nodes in *V* and edges in *E*, if node 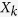 (edge 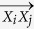) exists in 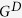 but not in 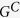, we treat 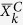 and the variance of 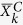 (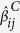 and the variance of 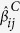) equal to zero, and vice versa. For instance, Fig. 1A describes the network structure generated from three branches of unfolded protein response (UPR) under sever ER stress^19^. The biological evidence is the three branches *ATF6, PERK* and *IRE1* can be activated when the chaperone *GRP78* is recruited to misfolded proteins accumulating in the ER. We imagine the nodes 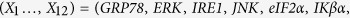 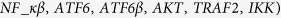, then the corresponding weight vector for these 12 nodes is 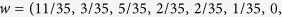

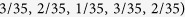
, while 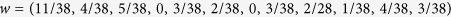 when the reverse direction between 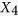 and 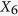 (Fig. 1B). If 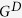 and 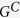 have the same structure as in Fig. 1A, then *K* = 12, *M* = 15. If 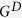 has structure as in Fig. 1A while 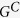 with structure as in Fig. 1B, then we treat *K* = 12, *M* = 16, 
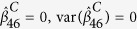
, 
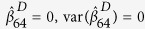
.

The idea behind our proposed statistic stems from that two nodes, even with the same magnitude of nodes differences, may still contribute unequally to the whole network difference because of the different relative position hiding in the topology structure. More weight has been put on the differences of nodes locating on relatively important position. The term 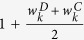 has been adopted to represent the relative importance for 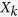, the intuition is that the baseline weight is one for difference of nodes without children, and additional magnitude 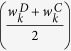 that represents the average relative weight should be added to the difference of nodes with some children. Alvo *et al*.^20^ have proposed a rank test (*RT*) which can distinguish significant changes due to either correlations or changes in the mean or both for group of genes in microarray experiments. For the *K* genes, it first subtracts the median expression value obtained from the combined case and control groups, from each gene expression value. This process aligns the data thereby inducing subsequent analyses to be sensitive to changes in the mean. Then, for the 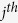 subject in group 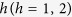, let 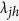 represent the vector of ranks of the aligned intensity values of the *K* genes. Let 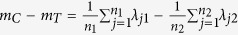, the rank test is defined as 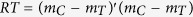, where the prime indicates the transpose of the vector.

We also compare the proposed statistic with its corresponding unweighted version *NES* and the statistic only considering nodes change *NS* and edges change *ES,* where


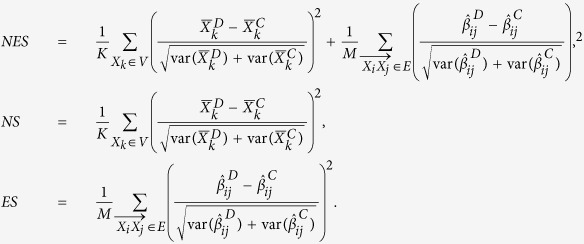


Our proposed method seems to be the linear combination of some chi-square statistics. The asymptotic theoretical properties have been explored for the linear combination of chi-square distributions under the framework of multivariate normal distribution^21^, especially for non-negative definite quadratic forms in non-central normal variables^22^. Nonetheless, it is nontrivial here to obtain the asymptotic distribution, since the covariance between the statistic of different nodes and different edges highly depend on the specific network structure. In other words, the asymptotic properties are network-specific. Meanwhile, it is also difficult to obtain the asymptotic distribution for *RT* test. To solve this problem, we adopted the strategy of a permutation test to get the empirical *P* value and assess the statistical significance^23^, which can be conducted as follows: (1) calculate the observed statistic from the original sample; (2) randomly re-assign subjects to one of two groups to get the permutation sample, while keeping the sample size for each group the same as the original observations; (3) perform the above steps many times (e.g. 1000) and calculate the statistic for each permutation sample; (4) obtain the *P* value as the proportion of permuted statistics greater than or equal to the observed one.

## Simulation

Simulations were designed to evaluate the type I error rate and statistical power, to compare the performance of *WNES, NES, NS, ES* and *RT* under different sample size and network structure. The statistical power is defined as the probability that the two biological networks are claimed to be different when the group difference of these two networks indeed exists. Based on the interplay network structure as in Fig. 1A, we first independently generate 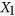 from 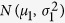, then 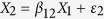, 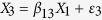, 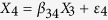, 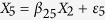, 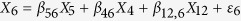, 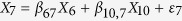, 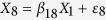, 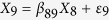, 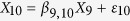, 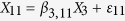, 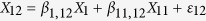, where 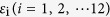 are the independent residual error terms. Under 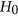, we assess the type I error rate under various sample sizes (100, 200, 300, 400, 500 for each group) given all the error terms follow 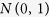 and the parameter setting 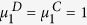, 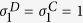, 
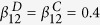
, 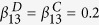, 
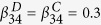
, 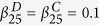, 
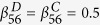
, 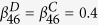, 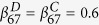 and other 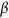 values equal to zero. Under 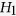, we designed four scenarios: (I) only node changes with 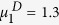, 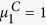; (II) only edge changes with 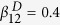, 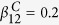; (III) changes of edge as in (II) and changes of node 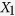 with 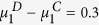 and 
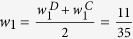
; (IV) changes of edge as in (II) and changes of node 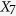 with 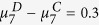 and 
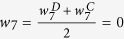
.

Note that under the above scenario (II), (III) and (IV), we must rectify the error term’s distribution to guarantee that all of the unchanged nodes have the identical distribution and all of the unchanged edges have the same magnitude between the two groups. Furthermore, to appraise the performance of these statistics to identify the changes in edge direction, we consider another situation when 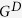 has structure (Fig. 1A) different from that in 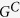 (Fig. 1B), Two scenarios are also designed: (I) only edge direction change with 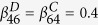 to evaluate the ability for detecting the direction difference; (II) only edge direction as above but treat 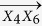 in 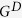 and 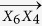 in 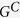 as the same edge, and compare 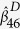 and 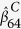 directly for *WNES, NE*S, NS, ES and *RT.*

**Figure 2:**
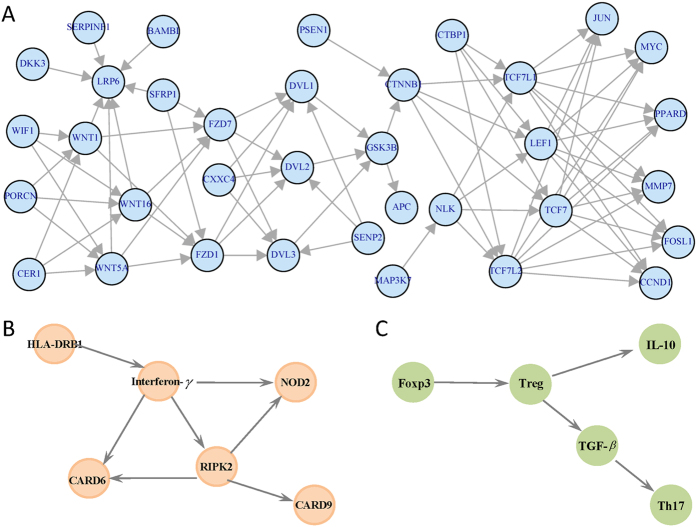
The analyzed network structure for gene expression data of lung cancer (A), GWAS data of leprosy (B) and acute myeloid leukemia data (C).

To evaluate the scalability of the proposed methods and to make the parameter setting more realistic, we also conduct another simulation based on one gene expression data from large airway epithelial cells sampled from 97 patients with lung cancer, 90 controls^24^,^25^. We focus on the 35 genes of *Wnt* canonical signaling pathway, the network structure is obtained from the KEGG database (Fig. 2A). Then in our simulation, the distribution of the nodes, the correlation between the nodes, and the magnitude about the changes in the nodes and the changes in the edges can be calculated based on this real data. We first calculated the sample mean differences of gene *CTNNB1* and *JUN* (−0.33 and 0.47, respectively), the difference of the edge linking *CTNNB1* and *PSEN1*(−0.28), between lung cancer patients and controls, and designed the following four scenarios: (I) only the node *CTNNB1* changes with magnitude equal to −0.33; (II) only edge linking *CTNNB1* and *PSEN1* changes with magnitude −0.28; (III) changes of edge as in (II) and changes of node as in (I), with relative weight for ***CTNNB1*** equal to 0.03; (IV) changes of edge as in (II) and changes of node *JUN* with magnitude 0.47 and the relative weight equal to 0.

A total of 1000 simulations were repeated for each sample size, and we permuted 1000 times for each configuration to assess the statistical significance by comparing the observed statistic with its empirical distribution.

### GWAS data of leprosy

A plausible biologic network underlying susceptibility to leprosy was created for depicting the functional relationship between some susceptibility genes identified from GWAS of leprosy^26^. The clustering of genotypes was carried out with the Gen-Call software version 6.2.0.4, which assigns a quality score to each locus and an individual genotype confidence score that is based on the distance of a genotype from the center of the nearest cluster. All the intensity-only SNPs and the SNPs on the X, Y and mitochondria chromosomes and the SNPs with call-rate lower than 90%, or MAF <1% in either cases or controls, or showing significant deviation from Hardy-Weinberg Equilibrium in the controls (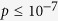), or having bad clusters were removed. From the initial GWAS data with 706 cases and 1225 controls, we only use the genetic matched 514 controls to minimize the effect of population stratification.

The original network includes genes *CARD6, HLA-DRB1, RIPK2, CARD9, interferon-γ, NOD2, PARK2, TNFSF15, LRRK2* and *NF-κB*. Since each gene contained several SNPs, we first calculated the first principal component (PC) with respect to all SNPs within one gene to represent the network node^27^. However, the SNP number within genes *PARK2, TNFSF15, LRRK2, NF-κB* are larger than sample size, thus we failed to conduct the PCA and we attempt to detect the difference between the networks including genes *CARD6, HLA-DRB1, RIPK2, CARD9, interferon-*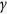 and *NOD2*.

All participants provided written informed consent, and the study was approved by the ethics committees of Shandong Academy of Medical Science^26^. The methods in this study were carried out in accordance with the approved guidelines. These 6 genes located on different chromosomes and totally contained 1119 SNPs (Supplementary Table S1), with network structure given in Fig. 2B.

### Acute myeloid leukemia data

Our acute myeloid leukemia (AML) data consisting of transcription factor forkhead box protein 3 (Foxp3), interleukin-10 (IL-10), T helper type 17 (Th17) cells, regulatory T (Treg) cells and their related cytokine transforming growth factor-beta (TGF-*β*) in bone marrow microenvironment from 23 AML patients and 7 controls collected by Qilu Hospital of Shandong University in China. Treg and Th17 are percentages, IL-10 and TGF-*β* are concentrations. When calculating Foxp3 quantities, β-actin transcripts were used as an internal control. Relative gene expression level of Foxp3 (the amount of target, normalized to endogenous control gene) was calculated using the comparative Ct method formula 2 −ΔCt. Therefore, there is no unit for Foxp3 quantity. AML patients were diagnosed based on the French-American-British (FAB) classification system. We excluded patients with hypertension, diabetes, cardiovascular diseases, chronic or active infection or pregnant. Individuals with slight iron deficiency anemia, having no immunological changes, were used as controls. The clinical characteristics of participants were provided in the Supplementary Table S2. The study was approved by the Medical Ethical Committee of Qilu Hospital, Shandong University, China. The methods in this study were carried out in accordance with the approved guidelines. Informed consent was obtained from all participants before enrollment in accordance with the Declaration of Helsinki. Th17 and TGF-β are significantly decreased, while Treg cells, related cytokine IL-10 and transcription factor Foxp3 were markedly elevated in AML patients compared to controls^28^. Some genes can present positive association, while others are negative. One interested thing is that whether their grouped network is associated with AML. The structure can be determined as follows (Fig. 2C), Foxp3 is essential for the development and function of Treg cells, Treg cells secrete IL-10 and TGF-β. And TGF-β, is the main regulator for Th17 differentiation^28^. We first scaled the data for *RT* test given that the nodes are different biological quantities with different units.

### Gene expression data of lung cancer

The proposed method was applied to a gene expression data set available on the GEO site (accession GDS2771), which is related to lung cancer. The expression data is from large airway epithelial cells sampled from 97 patients with lung cancer, 90 controls. The original study was approved by the Institutional Review Boards of all medical centers, and all participants provided written informed consent^24^,^25^. The methods were carried out in accordance with the approved guidelines. We focus on the 35 genes of *Wnt* canonical signaling pathway, the network structure is obtained from the KEGG database, totally 35 nodes and 79 edges are included (Fig. 2A). The probe sets corresponding to the same gene symbol were first averaged to obtain gene-level expression measurements.

## Results

### Simulation

Table 1 reveals that type I error rates of all five methods are close to nominal level 0.05 as a function of sample sizes, under the two network scenarios

**Figure 3:**
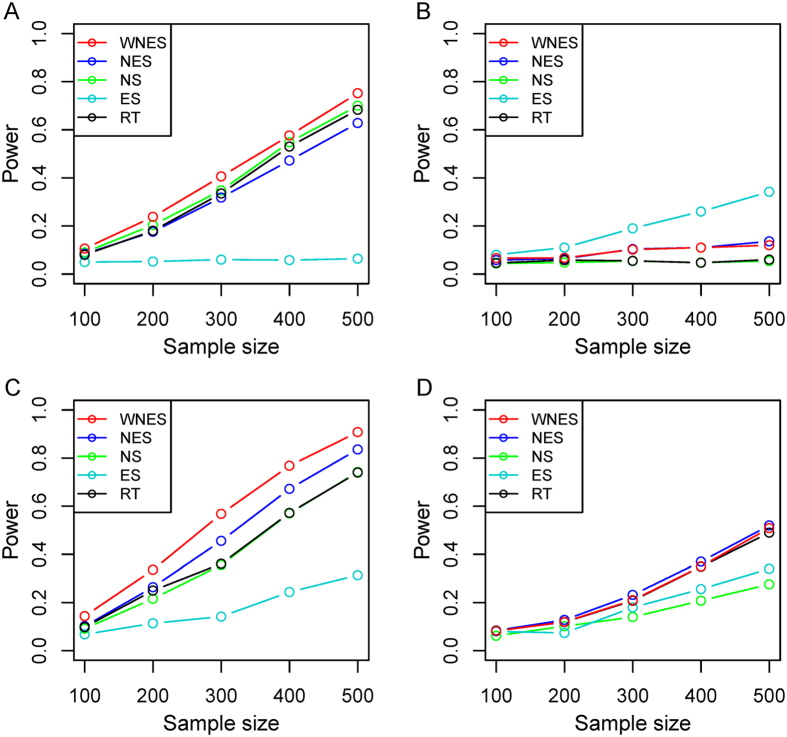
The power of the five statistics when two groups have same structure as in Fig. 1A, under the scenario only nodes change. (**A**) Only edge change (**B**), both nodes and edges change with the relative weight of the changing node greater than one (**C**), and both nodes and edges change with the relative weight of changing node equal to one (**D**).

Shown in Fig. 3 is the power when both 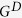 and 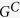 have the same structure as in Fig. 1A. Figure 3A shows the power when only the nodes change. As expected, *ES* has no power because it can only capture the edge change. *WNES* has a little higher power than that of *NS*, which is the gold standard in this case. Shown in Fig. 3B is the power when only the edge change, the power for *NS* vanished, *ES* expectedly presents the highest power, and the power for *WNES* and *NES* kept almost the same, though smaller than that of *ES*. No power can be found for *RT*, indicating that the correlation of these network node variables shows no difference between these two groups. Figure 3C illustrates the power when both the edges and nodes change, with the relative weight of the changed node greater than one, *WNES* shows the highest power. Figure 3D presents the power for the situation as in Fig. 3C except that the changing node has the relative weight equal to one. *WNES, NES* and *RT* have comparable and higher power than that of *NS* and *ES*.

**Figure 4:**
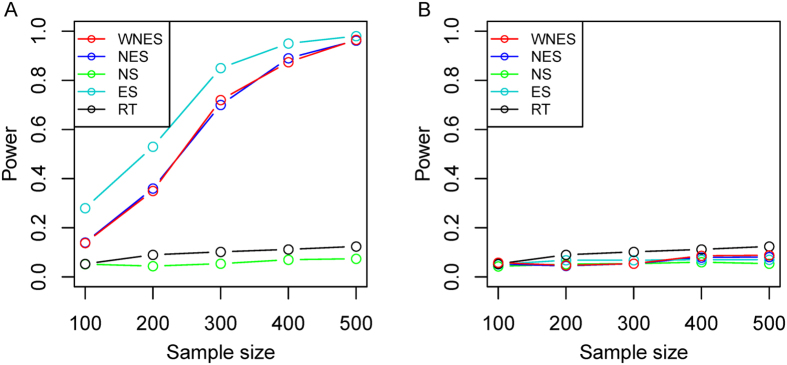
The power of the five statistics when two groups have different structure (Fig. 1A vs Fig. 1B), under the scenario only edge direction change (A), only edge direction change but treat 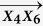 and 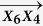 as the same edge (B).

Figure 4 demonstrates the power when 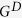 takes structure (Fig. 1A) different from that in 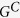 (Fig. 1B). Shown in Fig. 4A is the power when only edge direction change, *WNES* and *NES* still have almost the same ability to identify the direction change and show the relatively high power, though smaller than that of *ES*. If we ignore the direction difference, treating 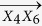 in 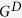 and 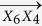 in 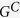 as the same edge and comparing them directly, then no power can be found (Fig. 4B) for all methods except *RT*, since there exists certain correlation changes for group of the network nodes due to the direction difference between 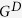 and 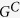. As expected, *RT* presents the same power as that in Fig. 4A.

**Figure 5:**
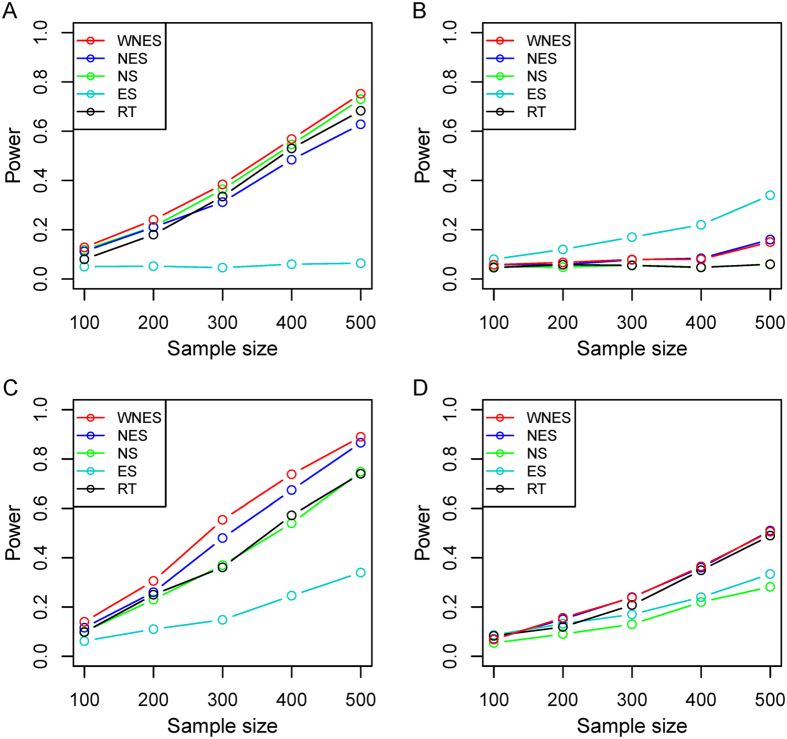
The power of the five statistics from another weight 
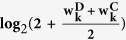
 under the same design as in Fig. 3.

Shown in Fig. 5 is the power with another weight, 
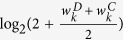
, under the same design as in Fig. 3, it indicates that WNES still has better performance.

**Figure 6:**
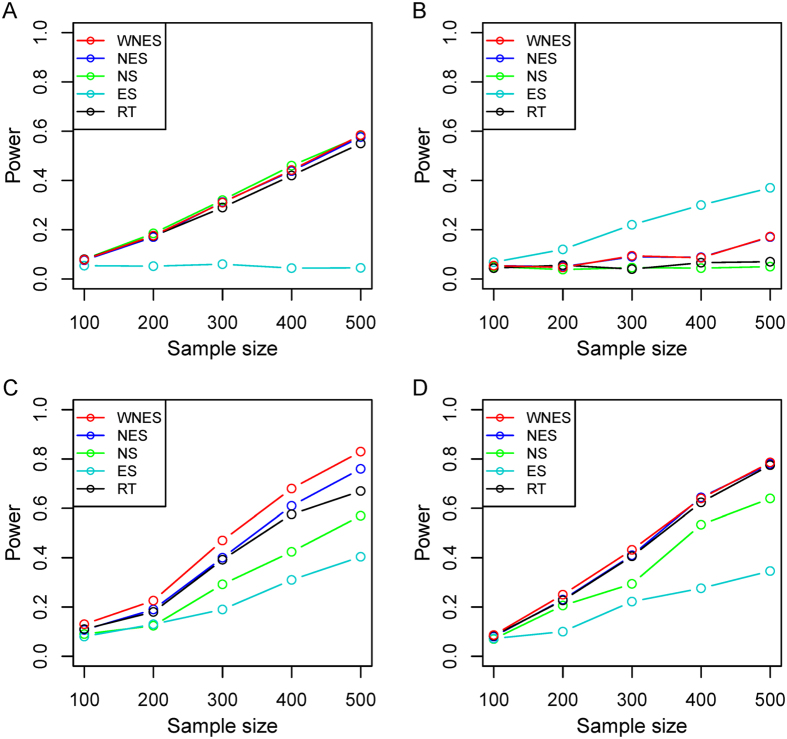
The power of the five statistics when two groups have the same structure as in Fig. 2A (35 nodes and 79 edges) under the same design as in Fig. 3.

Figure 6 shows the simulated results based on the real gene expression data of lung cancer, with network structure extracted from the *Wnt* signaling pathway. Similar phenomenon can be observed.

### Applications

For GWAS data of leprosy, all five methods except the *RT* and *ES* statistic can detect the network difference significantly (Table 2). The statistic *ES* only capturing the edges changes presents no significance, which may be partly due to that these 6 genes locate on different chromosome and have little correlation (Supplementary Table S1). All the network difference may be attributed to the node changes. For the AML data, simple Wilcoxon rank-sample test shows only IL-10 and TGF-*β* have the significant difference (Supplementary Table S2), while all five methods present significant network difference, though the edge changes statistic *ES* shows *P* value nearly 0.05. It seems that the network difference can be ascribed to both node and edge changes, and the *WNES* shows smaller *P* value than that of the other methods. For gene expression data of lung cancer, all methods except *ES* show significant network difference, and *WNES* shows smallest *P* value.

## Discussion

Numerous risk factors are woven into biological networks that dominate the disease occurrence, development and prognosis. The effect of one single factor can change substantially when put it within one network, or vice versa. From the perspective of systems medicine, different physiological conditions such as cases and controls manifest as different biological networks. Two sample statistical comparison between biological networks can provide not only new insight into the disease mechanism but also statistical guidance for drug development. Meanwhile, although the traditional epidemiology has successfully identified a list of risk factors, there still exist a black box from the exposures to the disease. Recent advances in high-throughput technologies allow a shift from the single paradigm to a new paradigm based on systems epidemiology, which aims to integrate putative lifestyle exposures and biomarkers, extracted from multiple omics platforms, to offer new insights into the network mechanisms underlying disease at the human population level. A key but inadequately addressed issue is to develop valid statistical method to test possible differences of the networks between two groups.

Bearing in mind that network difference can result from not only changes in the nodes but also changes in the edges (both the magnitude and direction), we proposed a novel statistic *WNES* for detecting the group difference between directed networks, accounting for network structure through putting more weights on the difference of nodes locating on relatively more important position, which was determined by the number of their own children nodes. Simulations showed that the proposed statistic was stable and had comprehensively better performance under various scenarios, except the case that only the edge change. The changes in biological network can be first attributed to changes in the nodes with a larger probability. Biologically, the change in the edge should be probably due to the changes in some nodes (linking this edge or not). On the other hand, the change in the node is statistically corresponding to the change of one moment of random variables, while the change in the edge is corresponding to the change of second moment of random variables, the calculation of the second moment usually depends on the one moment. Furthermore, decomposing the whole network difference into changes in the nodes and changes in the edges can help to interpret the whole network better. It naturally provides us whether the network difference is due to changes in the nodes or changes in the edges or both.

Network comparison for GWAS of leprosy and AML data further confirm that the proposed *WNES* have advantages in practice. All the network difference from GWAS data of leprosy may be attributed to nodes differences given that the 6 genes locate on different chromosome and thus have little correlation. This finding is consistent with the results reported earlier^21^, and provides the statistical evidence for gene interaction network obtained from an ingenuity pathways analysis. *HLA-DR* molecules present M. leprae peptide antigens to CD4+ T cells, which allows the T cells to be activated. In leprosy, this process is thought to lead to the generation of Th1 cells, which produce interferon-γ, resulting in macrophage maturation and the production of antimycobacterial molecules. Failure of this process is thought to be critical for susceptibility to leprosy and infection by other mycobacteria^29^. *NOD2* and *RIPK2* can be regulated by interferon-γ, which is consistent with the finding that persons with mutant interferon-γ are susceptible to mycobacterial infection^30^. *RIPK2* can regulate the *CARD* gene, and ligand bound to *NOD2* initiates signaling, can be also mediated by *RIPK2* through a ubiquination process that involves the recruitment of *TAK1* and *NEMO* to the *NOD2–RIPK2* complex^31^. The network difference of AML data can be owed to both the changes in nodes and changes in edges, Foxp3 was demonstrated to be exclusively expressed by Treg cells^32^, which mediate suppression in a cell contact-dependent manner or via cytokine-dependent pathways by releasing suppressor cytokines such as IL-10 and TGF-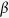
33. Also, it was reported that Treg-derived TGF-*β* actually promoted the development of Th17 cells^34^. For gene expression data of lung cancer, all methods except *ES* show significant network difference and *WNES* presents smallest *P* value. The role of *Wnt* signaling in lung cancer is well established^35^,^36^. Several *Wnt* proteins are differentially expressed in non–small cell lung cancer (NSCLC) specimens, for instance, *WNT1* is overexpressed in NSCLC samples, and cancer cells expressing *WNT1* are resistant to apoptotic therapies. The *WNT* regulator, *WIF*, as well as *SFRP1* and *DKK3*, are down-regulated in NSCLC due to transcriptional silencing via hypermethylation of their promoters. It has been illustrated that active *WNT* signaling in NSCLC is mediated by overexpression of the intracellular signal transducer, *DVL*. Specifically, *DVL3* was overexpressed in microdissected NSCLC samples, and inhibition of *DVL* decreased b-catenin expression and cell growth^37^.

The motivation to the weight is that two nodes, even with the same magnitude of nodes difference, may still contribute unequally to the whole network difference due to the different relative position hiding behind the topology structure. More weight should be put on the difference of nodes locating on relatively important position, which was represented by the number of child nodes (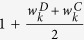). The intuition is that the baseline weight is one for difference of nodes without children, and the average of the number of child nodes should be added to the difference of nodes with some children. One important question is how to choose the appropriate weight to measure the strength that topological differences contribute to the overall network difference, we here introduce two optional user-adjustable weights (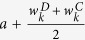) and 
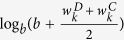
, where smaller *a* and *b* represent more contribution of topological differences. One limitation of the proposed test is that the theoretical property is difficult to obtain in its current form, thus lead to relatively high computation burden. Meanwhile, the loop regulation can be commonly encountered in biological networks, such as feedback loops, a circular chain of interaction, which can affect dynamical behaviors in the course of network evolution, particularly the robustness of a network^38^,^39^. In this case, the weight of a node determined by the number of daughter nodes is invalid, since it is difficult to capture the parent node when there is loops, other measures to characterize node importance in looped biological networks is highly desirable, and can be adopted to develop the loop version of the proposed test. The current method is limited to directed acyclic graph.

The proposed statistic can be treated as the extension for directed network of our recent study^40^. Little attention has been paid on the biological network structure learning problem. It needs to determine every possible edge with highest degree of data matching to constructing network structure, including whether the edge exists and which direction the edge orients. The network topology depends heavily on the structure learning algorithm. However, it is still of great significance to consider the case when the real network is unknown. Actually, most biologists often have a growing awareness of the interplay between the biological components and can depict more or less the specific network or pathway for the corresponding biological process. Meanwhile, numerous databases (e.g. KEGG, GO) can help us to further establish the network structure.

Recently, several approaches using network-information to score differences between groups have been proposed^41^,^42^,^43^, including methods that take both the network topology and scores for individual nodes into account and evaluate the predictive power of the scores for sample classification. For instance, Rapaport *et al*.^41^ have concluded introducing a priori knowledge of a gene network for gene expression data analysis leads to good classification performance. The main motivation of our manuscript is to develop a new statistic for detecting group difference of directed biological networks, which is independent of the nature of the network. Furthermore, one whole network or system can be decomposed into many specific subnetworks or pathways, we can use the proposed statistic to explore which pathway is most statistically significant. This may provide the statistical evidence to give the most significant pathway priority for potential drug development. It can also be utilized to identify whether one specifically functional pathway is responsible for the disease. Nevertheless, it is also great significance to use the associated network or pathway for classification, the key is how to integrate the whole directed pathway information into one score, which should retain the node, edge and direction information.

Statistical comparisons between biological networks are in great need in many disciplines. The proposed *WNES* is powerful to detect group difference between directed biological networks. Source R code for the proposed methods is available on our website (http://119.188.112.184:107/comparison.txt).

## Additional Information

**How to cite this article**: Yuan, Z. *et al*. A powerful weighted statistic for detecting group differences of directed biological networks. *Sci. Rep.* **6**, 34159; doi: 10.1038/srep34159 (2016).

## Supplementary Material

Supplementary Information

## Figures and Tables

**Table 1:** Type I error of the five statistics.

Sample size	200	400	600	800	1000
Network	**12 nodes and 15 edges**				
*WNES*	0.048	0.052	0.056	0.045	0.050
*NES*	0.046	0.049	0.042	0.053	0.056
*NS*	0.041	0.059	0.044	0.051	0.047
*ES*	0.055	0.047	0.053	0.051	0.057
*RT*	0.042	0.056	0.055	0.048	0.044
Network	**35 nodes and 79 edges**				
*WNES*	0.058	0.042	0.049	0.054	0.045
*NES*	0.048	0.044	0.053	0.046	0.047
*NS*	0.060	0.041	0.058	0.049	0.050
*ES*	0.059	0.046	0.055	0.043	0.053
*RT*	0.040	0.044	0.054	0.046	0.058

**Table 2:** *P*-values of the five statistics for the three real data sets.

	GWAS of Leprosy	AML Data	Lung Cancer Data
*WNES*	0.007	0.0016	0.0086
*NES*	0.012	0.0028	0.0125
*NS*	0.0003	0.0038	0.0209
*ES*	0.305	0.0471	0.0813
*RT*	0.142	0.0040	0.0228
