# Supplementary material for: A powerful weighted statistic for detecting group differences of directed biological networks
Source: Sci Rep. 2016 Sep 30;6:34159. doi: 10.1038/srep34159 (PMC5054825; doi:10.1038/srep34159)
Supplement: Supplementary Information [file srep34159-s1.doc]

**Supplementary Information**

**A powerful weighted statistic for detecting group differences of directed biological networks**

Zhongshang Yuan^1,*^, Jiadong Ji ^1,*^, Xiaoshuai Zhang^1^, Jing Xu^1^, Daoxin Ma^2^,

Fuzhong Xue^1,#^

^1^Department of Biostatistics, School of Public Health, Shandong University, Jinan 250012, China

^2^Department of hematology, Qilu hospital of Shandong University, Jinan 250012, China

^*^ These authors contributed equally to this work.

^#^Correspondence: xuefzh@sdu.edu.cn

Postal address: PO Box 100, Department of Biostatistics, School of Public Health, Shandong University; 44, Wenhua west Road, Jinan 250012, China.

Phone number: +86 13906405997

Fax number: +86 0531 88380280

**Table S1**. The location and SNP number for 6 susceptibility genes belonging to the network associated with leprosy

| Gene | Gene ID | SNP number | Chromosome |
| --- | --- | --- | --- |
| *CARD6* | 84674 | 197 | 5 |
| *HLA-DRB1* | 3123 | 246 | 6 |
| *RIPK2*  *CARD9*  *interferon-*$\gamma$  *NOD2* | 8767  64170  3458  64127 | 181  69  221  205 | 8  9  12  16 |

**Table S2. Clinical characteristics of subjects grouped according to AML status**

|  | All (*n*=30) | AML (*n*=23) | controls (*n*=7) | *P* value |
| --- | --- | --- | --- | --- |
| Age (year) | 38.60 ± 12.42 | 40.35±11.59 | 32.86±14.24 | 0.2377 |
| Gender (female %) | 16 (53.33%) | 11 (47.83%) | 5 (71.53%) | 0.3392 |
| Foxp3 | 0.0151(0.0153) | 0.0144 (0.0188) | 0.0199 (0.0057) | 0.2661 |
| Treg (%) | 2.27 (2.415) | 2.77 (4.205) | 1.35 (1.1604) | 0.2484 |
| IL-10 (pg/ML) | 3.064 (3.165) | 3.252(3.098) | 2.25(1.412) | 0.024 |
| TGF-$\beta$(pg/ML) | 3918.66 (7583.11) | 3131.66(3571.93) | 11488.58(6905.43) | 0.0002 |
| Th17 (%) | 2.595(2.618) | 2.33(1.931) | 3.61 (1.606) | 0.2455 |

*Note:* Data are presented as mean±SDs or median (IQRs), continuous variables are compared with two-sample t test or Wilcoxon rank-sum test, and categorical variables with Fisher-exact test.
